# Effects of Antimicrobial Photodynamic Therapy Mediated by Free Curcumin and Curcumin-Associated Superparamagnetic Iron Oxide Nanoparticles on *Candida* spp. and Microcosm Biofilms Associated with Denture Stomatitis

**DOI:** 10.1021/acsomega.6c04006

**Published:** 2026-10-02

**Authors:** Amanda Siqueira Fraga, Anna Carolina Pinheiro Lage, Karoline Kristina Kemmerich, Juliana Cin Fung Jang Bartachini, Felipe de Camargo Ribeiro, Maíra Terra Garcia, Juliana Campos Junqueira, Paulo Henrique Fonseca do Carmo

**Affiliations:** † Department of Biosciences and Oral Diagnosis, Institute of Science and Technology, 164767 São Paulo State University (Unesp), São José dos Campos, São Paulo 12245-000, Brazil; ‡ Department of Biotechnology Applied to Pathogens, René Rachou Research Centre, Fiocruz Minas, Belo Horizonte, Minas Gerais 30190-009, Brazil; § Special Mycology Laboratory, Paulista School of Medicine, 28105 Federal University of São Paulo (Unifesp), São Paulo, São Paulo 04023-062, Brazil; ∥ Department of Genetics, Microbiology and Immunology, Botucatu Institute of Biosciences, São Paulo State University (Unesp), Botucatu, São Paulo 18618-689, Brazil

## Abstract

Antimicrobial photodynamic therapy (aPDT) has emerged as a promising strategy for managing infections associated with*Candida*spp. In this context, nanoformulations of photosensitizers have been investigated to enhance the antimicrobial effects of established compounds, such as curcumin (CUR). In the present study, we evaluated the efficacy of aPDT mediated by curcumin-associated superparamagnetic iron oxide nanoparticles (CUR-SPION) and CUR against*Candida*spp. and complex heterotypic biofilms. Clinical samples were collected from the prosthesis surfaces and oral mucosa of 5 patients clinically diagnosed with denture stomatitis.*Candida*isolates were identified using Matrix-Assisted Laser Desorption/Ionization Time-of-Flight Mass Spectrometry (MALDI-TOF MS). CUR-SPION were synthesized via the coprecipitation method, allowing the incorporation and association of CUR with the nanoparticles during their formation. Subsequently, their size, morphology, stability, and surface charge were characterized. The isolates were then subjected to treatment with CUR and CUR-SPION, followed by aPDT using visible blue light (wavelength of 420 nm, power output of 1,250 mW, power density of 0.16 W/cm^2^, and energy density ranging from 0.79–12.78 J/cm^2^) in planktonic cultures, monotypic biofilms, and microcosm biofilms. *Candida*yeasts were detected in 3 patients and identified as*C. albicans*and*C. dubliniensis*. For both species, the minimum inhibitory concentration (MIC) of CUR was 64 μg/mL in the nonirradiated group and 4 μg/mL after aPDT. In contrast, CUR-SPION exhibited MIC values >64 μg/mL under all conditions tested. CUR-mediated aPDT decreased*Candida*cell viability at 4 μg/mL and within 2 h of treatment by 3.6 log_10_ CFU/mL for*C. dubliniensis*P1, 3.2 log_10_ CFU/mL for *C. albicans* P2, and 2.0 log_10_ CFU/mL for*C. dubliniensis*P3, while CUR-SPION-aPDT showed no effect on biofilm viability. CUR-mediated aPDT also significantly reduced the viability of microcosm biofilms, causing inhibitions (log_10_ CFU/mL) of 1.3 to 2.2 for streptococci, 1.4 to 3.1 for *mutans* streptococci, 1.5 to 2.2 for staphylococci, and 1.2 to 3.1 for yeast. In conclusion, CUR-mediated aPDT was effective against*C. albicans*and*C. dubliniensis*isolated from denture stomatitis, with an extended spectrum, targeting fungi and bacteria in microcosm biofilms. In contrast, CUR-SPION showed no antifungal activity against*Candida*under the conditions tested.

## Graphical abstract

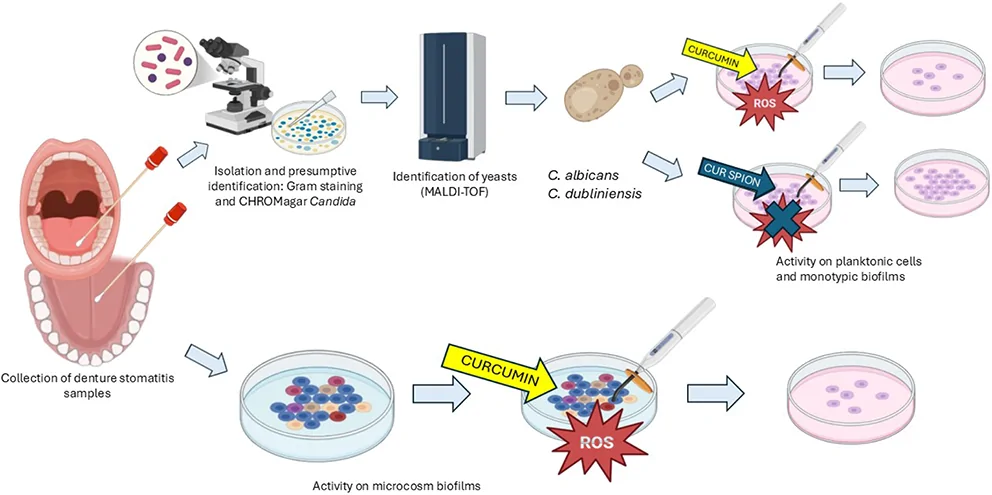

## Introduction

*Candida*spp. infections pose a significant public health challenge due to their high prevalence worldwide.^1^ *Candida*spp. can colonize several anatomical sites, including the oral cavity. Under normal conditions,*Candida*maintains a commensal relationship with its host. However, systemic and local factors can compromise this balance, leading to candidiasis.^2–4^ Candidiasis is the most common oral fungal infection, with denture stomatitis being one of its clinical manifestations. Denture stomatitis is an inflammatory condition of the oral mucosa, with a high prevalence, affecting 41% to 77% of denture users.^5–7^ This condition is usually related to trauma from ill-fitting or porous prostheses, prolonged denture use, inadequate hygiene, hyposalivation, and irritation or allergy caused by residual monomers released from acrylic dentures.^8,9^

In this manifestation,*Candida albicans*is the leading pathogen,^10,11^ acting in the development, persistence, and exacerbation of denture stomatitis. However, other species, such as*C. parapsilosis*,*C. dubliniensis*, and*C. tropicalis*, and species related to*Candida* spp., including*Pichia kudriavzevii*­(formerly known as*Candida krusei*) and *Nakaseomyces glabratus*­(formerly known as*Candida glabrata*), can also be present and contribute as co-infection agents.^12–14^

The pathogenicity of*Candida*spp. is largely attributed to their phenotypic plasticity and virulence factors. During infection, these yeasts can transition into filamentous forms such as pseudohyphae and hyphae, promoting tissue invasion. Moreover, the production of adhesins, proteases, and phospholipases, combined with their ability to form biofilms, enhances their capacity for tissue colonization and pathogenicity.^15,16^ Biofilms are highly structured microbial communities that shield microorganisms from antimicrobial agents, pH fluctuations, and nutrient limitations.^4^ Consequently, infections associated with biofilms are difficult to treat due to their reduced susceptibility to clinical antifungals.^17^

It is estimated that at least 80% of existing microorganisms reside within biofilms, highlighting the importance of understanding and investigating these complex structures.^18^ Microorganisms in biofilms exhibit distinct characteristics compared with their planktonic counterparts, including increased tolerance to antifungals, which contributes to the recurrence of fungal infections. In research, biofilms can be studied using *in vitro* or *in vivo* approaches.^16,19^ *In vitro* models offer controlled conditions but fail to fully replicate the physiological complexity of the host environment. In contrast, *in vivo* studies require well-defined experimental models that account for the host physiology and the surrounding microenvironment. In this scenario, microcosm biofilms offer an alternative model that bridges the gap between *in vitro* simplicity and *in vivo* complexity.^20,21^ These biofilms are established from biological material collected directly from the infection sites, thereby preserving the native biodiversity of the microbial community. They are typically composed of microorganisms from different species, effectively reproducing key features of the anatomical site, including microbial diversity, pH conditions, and ecological interactions.^20,22^

The limited efficacy of traditional antifungal therapies against biofilms, coupled with the emergence of antifungal-resistant strains and recurrent infections,^23,24^ highlights the urgent need for innovative antifungal strategies to address these challenges. In this context, antimicrobial photodynamic therapy (aPDT) has assumed a prominent role. aPDT operate through photodynamic processes involving nontoxic photosensitizers, molecular oxygen, and a light source. The photosensitizer, in the presence of a light source at a wavelength compatible with its absorption band, transitions from the ground state (singlet) to an excited state and then can undergo conversion to the triplet state, which is more stable and reactive. Once stabilized, the long-lived excited triplet state can participate in two types of photodynamic reactions—Type I and Type II, as summarized in Figure 1. In Type I reactions, the photosensitizer in its excited state interacts directly with biomolecules, such as lipids, proteins, or nucleic acids, through electron or hydrogen transfer. This process leads to the formation of free radicals, which subsequently react with molecular oxygen to generate a cascade of reactive oxygen species (ROS). In Type II reactions, energy is transferred directly from the excited triplet photosensitizer to molecular oxygen in its ground state, producing singlet oxygen. Both can occur simultaneously in aPDT, and the predominance depends on factors such as the type of photosensitizer, the oxygen concentration, and the characteristics of the cellular microenvironment. The ROS generated by these reactions interact with microbial proteins, lipids, and nucleic acids, resulting in protein denaturation, lipid peroxidation of the cell membrane, DNA damage, and altered gene expression. These changes impair the enzymatic activity of the antioxidant system and ultimately lead to microbial death.^25,26^

**Figure 1:**
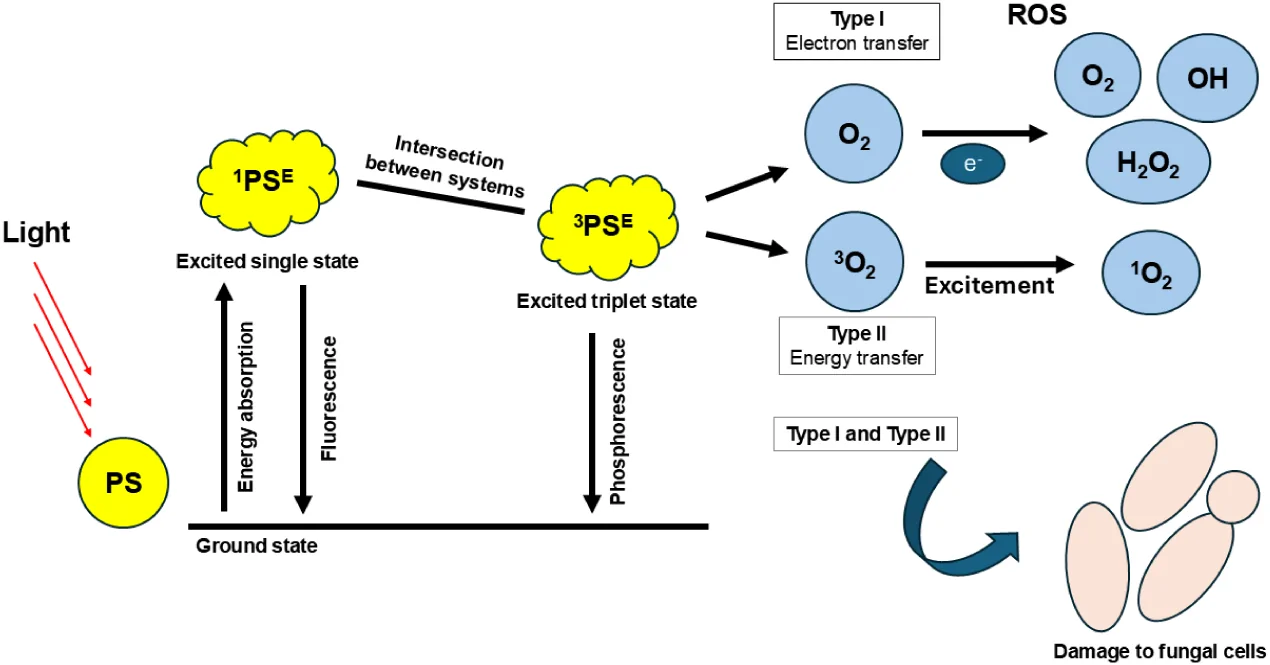
Diagram illustrating the photodynamic processes involved in antimicrobial photodynamic therapy (aPDT). Upon light irradiation, the photosensitizer (PS) is excited to the singlet state. After undergoing intersystem crossing, the PS reaches the triplet state, where it can follow two pathways: Type I reaction, by interacting with molecular oxygen to generate reactive oxygen species (ROS), or Type II reactions, by transferring energy to produce singlet oxygen. Ultimately, these reactive molecules can interact with fungal cell structures and organelles, leading to cell damage and fungal death. ^1^PS_E_: singlet state of photosensitizer, ^3^PS_E_: triplet state of photosensitizer, ^3^O_2_: triplet state of oxygen, ^1^O_2_: singlet state of oxygen.

Among photosensitizers, curcumin (CUR) is recognized for its established antimicrobial activity in aPDT,^27–30^ showing efficacy against planktonic cells, monotypic*Candida*biofilms,^31,32^ and dual-species biofilms.^33^ However, limited data are available regarding the impact of CUR-mediated aPDT on complex biofilms, particularly microcosmic biofilms that more closely resemble clinical conditions. Although CUR exhibits favorable *in vitro* and *in vivo* biocompatibility, its hydrophobic nature compromises bioavailability and restricts clinical application.^34,35^ To overcome these limitations, nanoformulation strategies were explored.

Nanoparticles have proven to be effective in enhancing the targeted delivery of therapeutic agents by prolonging circulation time and improving compound accumulation at the infection site.^36,37^ In addition, previous studies have demonstrated the antifungal effects of nonfunctionalized iron nanoparticles and iron nanoparticles conjugated with antifungals against *Candida* spp.^38–40^ However, the use of these nanoparticles as carriers for photosensitizers, such as curcumin, has been a recent approach. The use of superparamagnetic iron oxide nanoparticles (SPIONs) as carriers for CUR in aPDT combines physicochemical and biological properties that can enhance the therapeutic efficacy. This nanostructured system significantly improves CUR dispersion in aqueous media, increasing its stability, and facilitates delivery to the therapeutic target.^41–43^ In addition, these nanosystems have demonstrated low cytotoxicity in the absence of light (dark effect) but high photodynamic activity upon irradiation, highlighting their selective therapeutic potential.^44^ Furthermore, SPIONs promote cellular uptake of the photosensitizer and may act synergistically in the generation of ROS, thereby enhancing the cytotoxic effect of aPDT.^45^

Although studies have demonstrated the photodynamic effects of nanoparticle formulations on microcosm biofilms,^46,47^ the effects of curcumin-mediated aPDT on these structures remain poorly understood.^48^ Importantly, the use of CUR associated with superparamagnetic iron oxide nanoparticles as photosensitizers in aPDT has not yet been investigated in microcosmic biofilms. In this context, we synthesized and characterized curcumin-associated superparamagnetic iron oxide nanoparticles (CUR-associated SPION),^37,49–52^ and subsequently evaluated the effects of aPDT mediated by free CUR and CUR associated with superparamagnetic iron oxide nanoparticles on*Candida*spp. and complex heterotypic biofilms associated with denture stomatitis.

## Materials and Methods

### Experimental Design

This study was conducted in three stages. In stage 1, microcosm biofilm samples associated with denture stomatitis were collected from patients,^53^ transported to a microbiological analysis laboratory, and analyzed using Gram staining^54^ and cultivation in mycological media^55^ [Chromagar *Candida* Plus and Sabouraud dextrose agar (SDA)] to detect yeasts. Yeasts from positive samples were then isolated and identified via matrix-assisted laser desorption/ionization time-of-flight mass spectrometry (MALDI-TOF MS).^56^ In stage 2, the effects of antimicrobial photodynamic therapy (aPDT) mediated by free curcumin (CUR-aPDT) and curcumin-associated superparamagnetic iron oxide nanoparticles (CUR-SPION-aPDT) were assessed on*Candida*planktonic cells and monotypic biofilms through minimum inhibitory concentration (MIC) assay,^57^ time-kill curve analysis,^55^ and biofilm viability.^55^ In stage 3, the impact of CUR-aPDT on polymicrobial and complex biofilms^22^ was studied using the microcosm samples to determine microbial viability after CUR-aPDT treatment (Figure 2).

**Figure 2:**
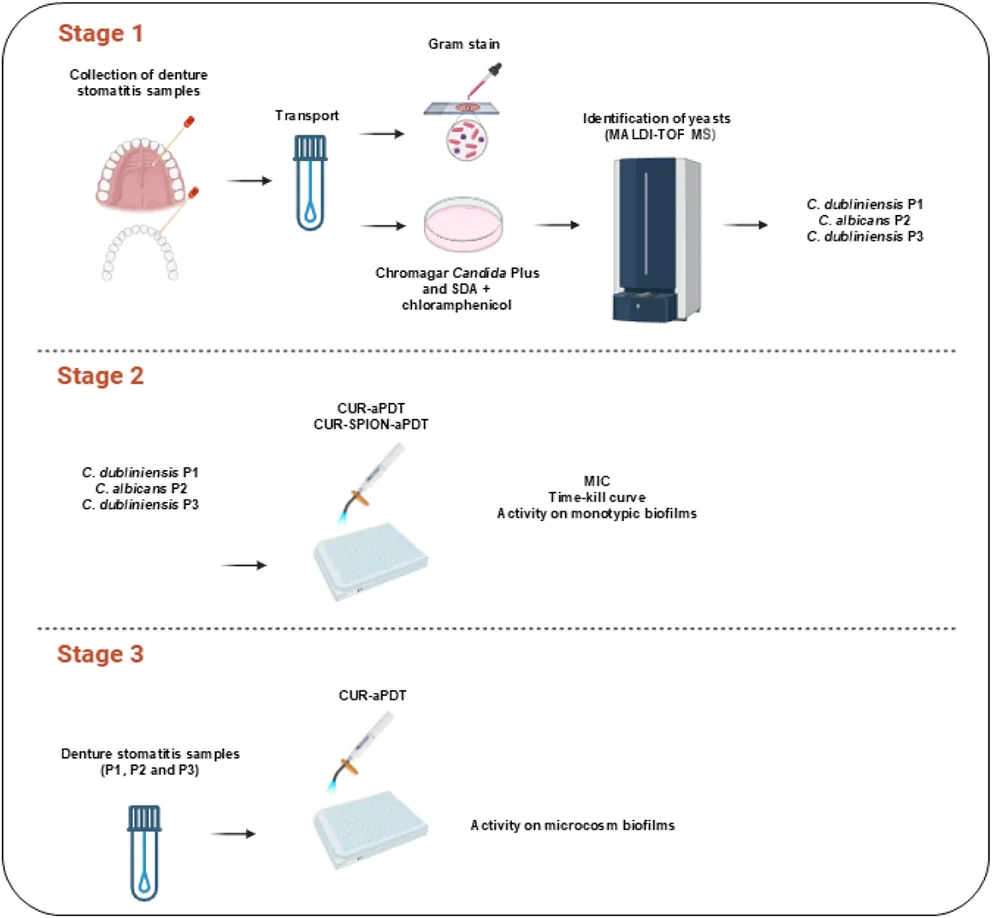
Diagram illustrating the stages outlined in the experimental design of this study. In Stage 1, samples of denture stomatitis were collected, transported to a microbiology laboratory, and analyzed using Gram staining and cultivation on both Chromagar*Candida*Plus and Sabouraud dextrose agar to detect yeasts. Yeasts were then isolated and identified using Matrix-Assisted Laser Desorption/Ionization Time-of-Flight Mass Spectrometry (MALDI-TOF MS), resulting in three identified isolates:*Candida dubliniensis*P1,*Candida albicans*P2, and*C. dubliniensis*P3. In Stage 2, the effects of antimicrobial photodynamic therapy (aPDT) mediated by free curcumin (CUR-aPDT) and curcumin-associated superparamagnetic iron oxide nanoparticles (CUR-SPION-aPDT) were assessed on*C. dubliniensis*­(P1 and P3) and*C. albicans*­(P2) planktonic cells and monotypic biofilms through minimum inhibitory concentration (MIC), time-kill curve, and biofilm viability assays. In Stage 3, the impact of CUR-aPDT on polymicrobial and complex biofilms from denture stomatitis samples (P1, P2, and P3) was evaluated using microcosm samples to determine microbial viability after CUR-aPDT treatment. Image created using BioRender (www.biorender.com).

### Ethical Aspects

The experimental protocol was approved by the Human Research Ethics Committee (CEPh) of the Institute of Science and Technology of Universidade Estadual Paulista (ICT/UNESP), São José dos Campos, under approval number 5.827755. The samples were collected at a Basic Health Unit in São José dos Campos, SP. The inclusion criteria were individuals using either complete dentures or removable partial dentures, aged 18 years or older, with no upper age limit, and no restrictions based on socioeconomic status, ethnicity, or gender. Individuals who had used antibacterials, antifungals, or corticosteroids within 60 days prior to sample collection were excluded.

### Sample Collection and Characterization

Five volunteers clinically diagnosed with oral candidiasis associated with dentures were included in this study. Samples were collected using cervical brushes both from the lesion on the palate and the dentures. The collected samples were stored in conical tubes containing sterile brain heart infusion broth (BHI, Kasvi, Paraná, Brazil) and transported to the Microbiology and Immunology Laboratory at ICT/UNESP up to 3 h after collection. Biological samples were transported by the researcher (DDS. Amanda Siqueira Fraga) at room temperature using triple packaging, specific documentation, and biosafety conditions in accordance with Resolution RDC no. 504/2021 of the Brazilian Health Regulatory Agency (ANVISA), and following the ethical guidelines established by Resolution no. 441/2011 of the Brazilian Health Council of the Ministry of Health.

Then, 100 μL of each sample were plated both on Sabouraud dextrose agar (SDA; HiMedia, Maharashtra, India) with 0.5% chloramphenicol and a chromogenic selective culture medium (Chromagar*Candida*Plus; Difco, New Jersey, USA) for presumptive identification of*Candida*. Following incubation for 24 h at 37 °C, the presence of creamy, smooth, shiny, elevated colonies with coloration ranging from white to creamy yellow on SDA, as well as colonies grown on Chromagar*Candida*Plus, was considered indicative of*Candida*spp. growth.^58,59^

Next, the presence of yeasts suggestive of*Candida*ssp. in the collected samples was confirmed by observing distinct microscopic fields of the Gram-stained smear at 1,000× magnification. Round or slightly oval cells, isolated or in groups, that stained Gram-positive-like were considered indicative of*Candida*spp.^54^

### Identification of Samples by Mass Spectrometry (MALDI-TOF MS)

Samples positive for*Candida*spp. were subjected to further identification. Colonies from the Chromagar*Candida* Plus plates were isolated by using the streak depletion technique on SDA with 0.5% chloramphenicol. The pure colonies obtained were identified using the MALDI-TOF MS Biotyper Microflex LT/SH system with the MBT Compass database (version 4.1.100; Bruker, Massachusetts, USA). Biotyper analyses are classified according to the following scores: values between 2.300 and 3.000 indicate reliable species identification; values between 2.000 and 2.299 indicate reliable genus and probable species identification; values between 1.700 and 1.999 indicate probable genus identification; and values below 1.700 indicate no reliable identification.^3^

### Curcumin-Associated Superparamagnetic Iron Oxide Nanoparticles Preparation

Curcumin-associated superparamagnetic iron oxide nanoparticles (CUR-SPION) were prepared by the coprecipitation method,^49,52^ with modifications.^37^ Ferric chloride (FeCl_3_·6H_2_O; 0.1 M) and iron­(II) chloride (FeCl_2_·4H_2_O; 0.05 M) were dissolved in deionized water, after which CUR (5 mM) was slowly added to the mixture under vigorous heating. The reducing agent ammonium hydroxide (NH_4_OH; 2 M) was then added until an alkaline pH (∼10) was reached and maintained under continuous heating for 30 min. The mixture was subsequently heated to 70 °C for 2 h and cooled to room temperature. The resulting CUR-SPIONs were washed three times with methanol and three times with ultrapure water to remove free curcumin, residual reagents, and organic solvents. The final CUR-SPION solution was prepared in ultrapure water.

### Characterization of Curcumin-Associated Superparamagnetic Iron Oxide Nanoparticles

The morphology and size of CUR-SPION were characterized by transmission electron microscopy (TEM; SpiritBiotwin FEI, 120 kV). The average nanoparticle size was calculated based on TEM images of at least 300 particles.^51^ The polydispersity index and zeta potential of CUR-SPION were determined by dynamic light scattering (DLS) using a Litesizer 500 instrument (Anton Paar).^51^ The functional groups present in CUR-SPION were investigated by Fourier transform infrared spectroscopy (FTIR; FTIR-8400S spectrometer, Shimadzu).^50^

### Photosensitizers and Light Source

CUR-SPION and curcumin (CUR) (Sigma-Aldrich, Missouri, USA) diluted in dimethyl sulfoxide (DMSO; Sigma-Aldrich) were used as photosensitizers. Control group with DMSO was included, and all experiments were conducted away from ambient light. The light source used was a blue light-emitting diode (LED; Emitter G, Schuster), emitting light at a wavelength of 420 nm^60^ with a power output of 1,250 mW, a power density of 0.16 W/cm^2^, and an energy density ranging from 0.79 to 12.78 J/cm^2^.^61^ After adding CUR, all plates were incubated for a preirradiation time of 5 min.^62^ An irradiation time of 40 s was selected based on preliminary experiments to determine the irradiation time in which significant antifungal effects were observed.

### Susceptibility Assay

The susceptibility of isolated*Candida*strains to CUR-SPION and CUR was determined using the broth microdilution method according to manual E.Def 7.3.1 of the European Committee on Antimicrobial Susceptibility Testing (EUCAST),^57^ with adaptations^61^ for aPDT application. We used concentrations ranging from 0.50 to 512 μg/mL for CUR, from 0.06 to 64 μg/mL for CUR-SPION, and from 0.03 to 32 μg/mL for fluconazole (FCZ; Sigma-Aldrich).

*Candida*spp. cultures previously grown for 24 h at 37 °C were suspended in saline, adjusted spectrophotometrically, and diluted in Roswell Park Memorial Institute (RPMI-1640) medium (Sigma-Aldrich; with l-glutamine, 2% glucose, and NaHCO_3_, buffered to pH 7.0 with 0.165 M 3-[N-morpholino]­propanesulfonic acid) to reach a final concentration of (1–5) × 10^5^ cells/mL. Microplates were then inoculated with 100 μL of the adjusted inoculum and incubated at 37 °C for 48 h. Growth control, sterile control, and quality control (using the strain*P. kudriavzevii*ATCC 6258) were included. The experiment was conducted with both an irradiated and nonirradiated group using the established parameters. The minimum inhibitory concentration (MIC) was considered as the lowest concentration capable of inhibiting 90% of fungal growth for CUR-SPION and CUR, and 50% for FCZ, compared to the growth control.

### Time-Kill Curve

Time-kill experiments were conducted using RPMI-1640 with 2% glucose as the growth medium. Based on the irradiated MIC values of CUR and the maximum concentration tested of CUR-SPION (64 μg/mL), isolated*Candida*strains (2.5 × 10^5^ cells/mL) were treated with CUR-SPION and CUR and subsequently irradiated using the established parameters of aPDT. Aliquots were taken at 0, 2, 8, and 24 h after exposure and plated on SDA to determine the colony-forming units (CFU/mL), corresponding to fungal viability following treatment for each experimental group.^55^ An untreated control group (C-L-) and groups treated with CUR-SPION (CS+L-) and CUR (C+L-) without irradiation were included.

### Antimicrobial Photodynamic Therapy Mediated by CUR-SPION and CUR on Monotypic Biofilms from Isolated *Candida*spp

Biofilm formation of the isolated*Candida*strains was performed as previously described.^63^ Monotypic biofilms were formed at the bottom of the 96-well microplates from*Candida*cultures previously grown for 24 h at 37 °C.*Candida*isolates were suspended in saline solution, quantified using a hemocytometer, and adjusted to 10^7^ cells/mL in yeast nitrogen base (YNB; HiMedia) supplemented with 100 mM glucose. An aliquot of 200 μL of the fungal suspension was then added to each well, and the microplates were incubated for 90 min at 37 °C under 75 rpm. Subsequently, the wells were washed twice with PBS, and 200 μL of YNB broth was added, followed by incubation at 37 °C for 48 h. After that, mature biofilms were treated with CUR-SPION at 1× and 5× MIC, and with CUR at 1×, 5×, and 10× MIC. Due to limitations in the maximum achievable concentration of CUR-SPION and its elevated MIC values, it was not possible to assess the effects of CUR-SPION at 10× MIC. An untreated control group (C-L-) and groups treated with CUR-SPION (CS+L) and CUR (C+L-) without irradiation were included. Subsequently, the plates designated for light exposure were irradiated, while the others remained in darkness. After this period, biofilms were disrupted using an ultrasonic homogenizer (Sonoplus HD2200, Bandelin Electronic, Berlin, Germany) with a power of 7 W for 30 s. The resulting suspension was plated on SDA, and the plates were incubated for 24 h at 37 °C to determine fungal viability (CFU/mL).^55^

### CUR-Mediated Photodynamic Therapy on Microcosm Biofilms from Denture Stomatitis

Microcosm biofilms were formed on sterile acrylic resin specimens (5 mm in diameter, 2.5 mm in thickness, and a surface area of 0.38 cm^2^) composed of poly­(methyl methacrylate) (Vipi Cril Plus, VIPI, São Paulo, Brazil) as previously described.^22^ Microcosm biofilm samples stored at −80 °C were thawed and homogenized. An aliquot (400 μL) was transferred to 20 mL of BHI broth supplemented with 5% sucrose, followed by incubation at 37 °C in 5% CO_2_ for 48 h. The suspension was centrifuged, washed twice with PBS, and resuspended in 20 mL of PBS. Subsequently, 225 μL of this microbial suspension was added to each well of 24-well microplates containing sterile acrylic resin specimens in 2 mL of BHI broth with 5% sucrose. The microplates were then incubated for 120 h at 37 °C in 5% CO_2_ to allow biofilm formation. Wells were washed twice with PBS, and 2 mL of fresh BHI broth with 5% sucrose was replaced every 24 h. Then, the specimens were treated with CUR at 10× MIC (40 μg/mL) without light (C+L-) and with light (C+L+). The concentration of 40 μg/mL was selected based on the results of CUR-mediated aPDT on monotypic biofilms. Since CUR-SPIONs did not exhibit antifungal activity against*Candida*strains under either dark conditions or as a photosensitizer in aPDT, subsequent assays were conducted using only CUR as the photosensitizer.

An untreated control group (C-L-) and a light-treated group (C-L+) were included. Following irradiation at the established parameters, the specimens were transferred to conical tubes containing 4 mL of saline solution, and the adhered biofilms were disrupted with a power of 7 W for 30 s. From the solution obtained, serial dilutions were prepared and plated in five different culture media: BHI for nonselective counting, Mitis Salivarius agar (MS; Difco) for counting streptococci, Mitis Salivarius Bacitracin Sucrose agar (MSBS; Difco) for counting for *mutans* streptococci, Mannitol agar (Difco) for counting staphylococci, and SDA with chloramphenicol for counting yeasts.^61,64^ The plates were then incubated at 37 °C for 24 h under 5% CO_2_ to determine microbial viability (CFU/mL).

### Statistical Analysis

The results obtained from the viable cell counts (CFU/mL) were normally distributed. For this reason, they were analyzed by analysis of variance (ANOVA), followed by Tukey test, using GraphPad Prism 5.0 software (GraphPad Software, Inc., San Diego, CA, USA). A minimum of four technical replicates per group were used for statistical analysis. The tests were repeated at least two times. A 5% significance level was adopted for all analyses.

## Results

### CUR-SPION Characterization

TEM analysis of CUR-SPION revealed a nanocomposite with a rounded morphology and darker regions corresponding to the iron oxide (Fe_2_O_3_) core. The images also indicated a tendency of the nanoparticles to agglomerate and form clusters (Figure 3A). Both TEM and DLS measurements showed an average particle diameter of 11.00 ± 2.0 nm (Figure 3B). FTIR analysis demonstrated the presence of CUR on CUR-SPION. The IR spectra displayed characteristic stretching vibrations at 1277, 1427, and 1628 cm^–1^, along with a prominent band at 1512 cm^–1^. Broad stretching vibrations were observed between 3200 and 3500 cm^–1^, and addition peaks appeared at 440 and 600 cm^–1^ (Figure 3C). The zeta potential of CUR-SPION was measured at −31.81 ± 0.38 mV.

**Figure 3:**
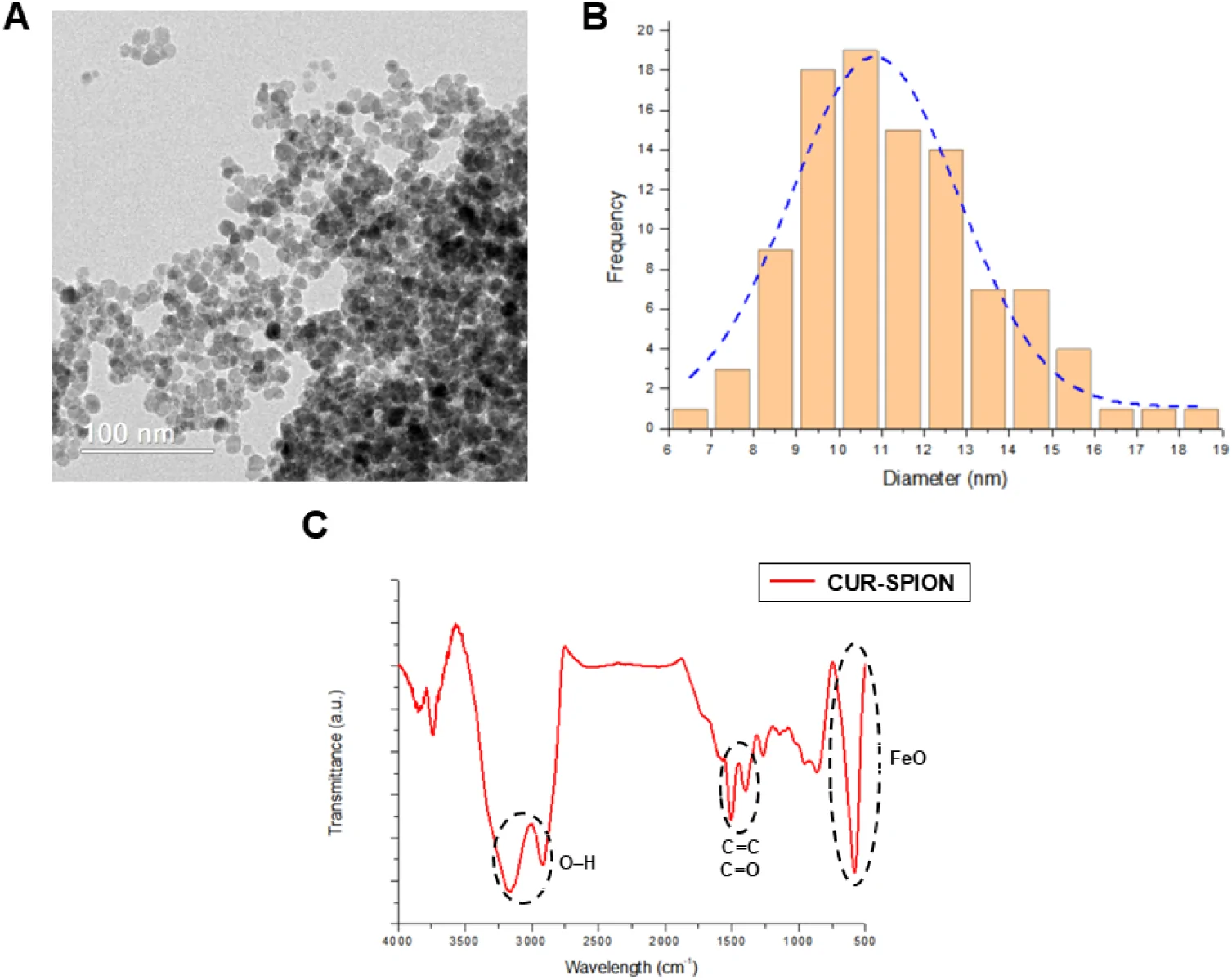
Characterization of curcumin-associated superparamagnetic iron oxide nanoparticles (CUR-SPION). A) Transmission electron microscopy (TEM) images of CUR-SPION. B) Size distribution histogram of 300 randomly selected nanoparticles measured from different clusters. C) Fourier-transform infrared (FTIR) spectra of CUR-SPION with key absorption bands highlighted.

### Isolation and Identification of*Candida*spp.

Initial slides prepared to check for yeasts suggestive of*Candida*, along with cultures grown on Chromagar*Candida*Plus medium, revealed*Candida*spp. in three of the five samples, designated P1, P2, and P3. Further identification demonstrated that the isolates from samples P1 and P3 were*C. dubliniensis*, while the isolate from sample P2 was identified as*C. albicans*­(score higher than 2.0) (Table 1).

**Table 1:** MALDI-TOF MS Identification of*Candida*Isolates Obtained from Samples of Patients with Denture Stomatitis

Isolate	Organism (best match)	Score
**P1**	*Candida dubliniensis*	2.122
**P2**	*Candida albicans*	2.497
**P3**	*Candida dubliniensis*	2.205

### aPDT Reduced MIC Values of CUR

The MIC value of CUR for the nonirradiated (NI) plate was 64 μg/mL against all strains. For the irradiated (IR) plates, it was 4 μg/mL of CUR for all stased on the ratio between NI and IR plates, we observed a 16-fold reduction in MIC values following CUR-mediated aPDT compared to NI plates. The MIC of CUR-SPION exceeded 64 μg/mL under both NI and IR conditions. MIC values of FCZ were 0.25 μg/mL for*C. dubliniensis*P1 and*C. albicans*P2, and 0.12 μg/mL for*C. dubliniensis*P3 (Table 2).

**Table 2:** Minimum Inhibitory Concentration (MIC; μg/mL) Values of Curcumin (CUR), Curcumin-Associated Superparamagnetic Iron Oxide Nanoparticles (CUR-SPION), and Fluconazole (FCZ) for Irradiated (IR) and Nonirradiated (NI) Plates against*Candida*spp.^a^

**MIC**(μg/mL)					
	**IR**		**NI**		
Strains	CUR	CUR-SPION	CUR	CUR-SPION	FCZ
*Candida dubliniensis* (P1)	4 (16×)	>64	64	>64	0.25
*Candida albicans* (P2)	4 (16×)	>64	64	>64	0.25
*Candida dubliniensis* (P3)	4 (16×)	>64	64	>64	0.12

a Values in parentheses represent the reduction in CUR MIC observed on the irradiated plate (IR) compared to the non-irradiated plate (NI).

### CUR-Mediated aPDT Reduced Viability of*Candida*spp.

Kill curve assay analysis revealed that, while CUR-SPION-mediated aPDT did not impact fungal viability, CUR-mediated aPDT reduced viability at all evaluated time points across all*Candida*strains studied (Figure 4). Treatment with CUR without light (C+L-), CUR-SPION without light (CS+L-), and CUR-SPION with light (CS+L+) had no impact on the viability of*C. dubliniensis*P1,*C. albicans*P2, and*C. dubliniensis*P3. In contrast, aPDT (C+L+) reduced the viability of*C. dubliniensis*P1 at 3.6 log_10_CFU/mL at 2 h, 4.2 log_10_CFU/mL at 8 h, and 3.6 log_10_CFU/mL at 24 h compared to the C+L- group (Figure 4A). CUR-mediated aPDT (C+L+) also decreased the viability of*C. albicans*P2 at 2 (3.2 log_10_ CFU/mL), 8 (3.8 log_10_ CFU/mL), and 24 h (1.4 log_10_ CFU/mL) compared to C+L- (Figure 4B). Compared to the C+L- group, aPDT (C+L+) reduced the viability of*C. dubliniensis*P3 at 2 log_10_ CFU/mL, 2.9 log_10_ CFU/mL, and 0.6 log_10_ CFU/mL at 2, 8, and 24 h, respectively (Figure 4C). For all strains tested, a marked reduction in viability was observed at 8 h, followed by a significant increase in fungal counts at 24 h (Figure 4).

**Figure 4:**
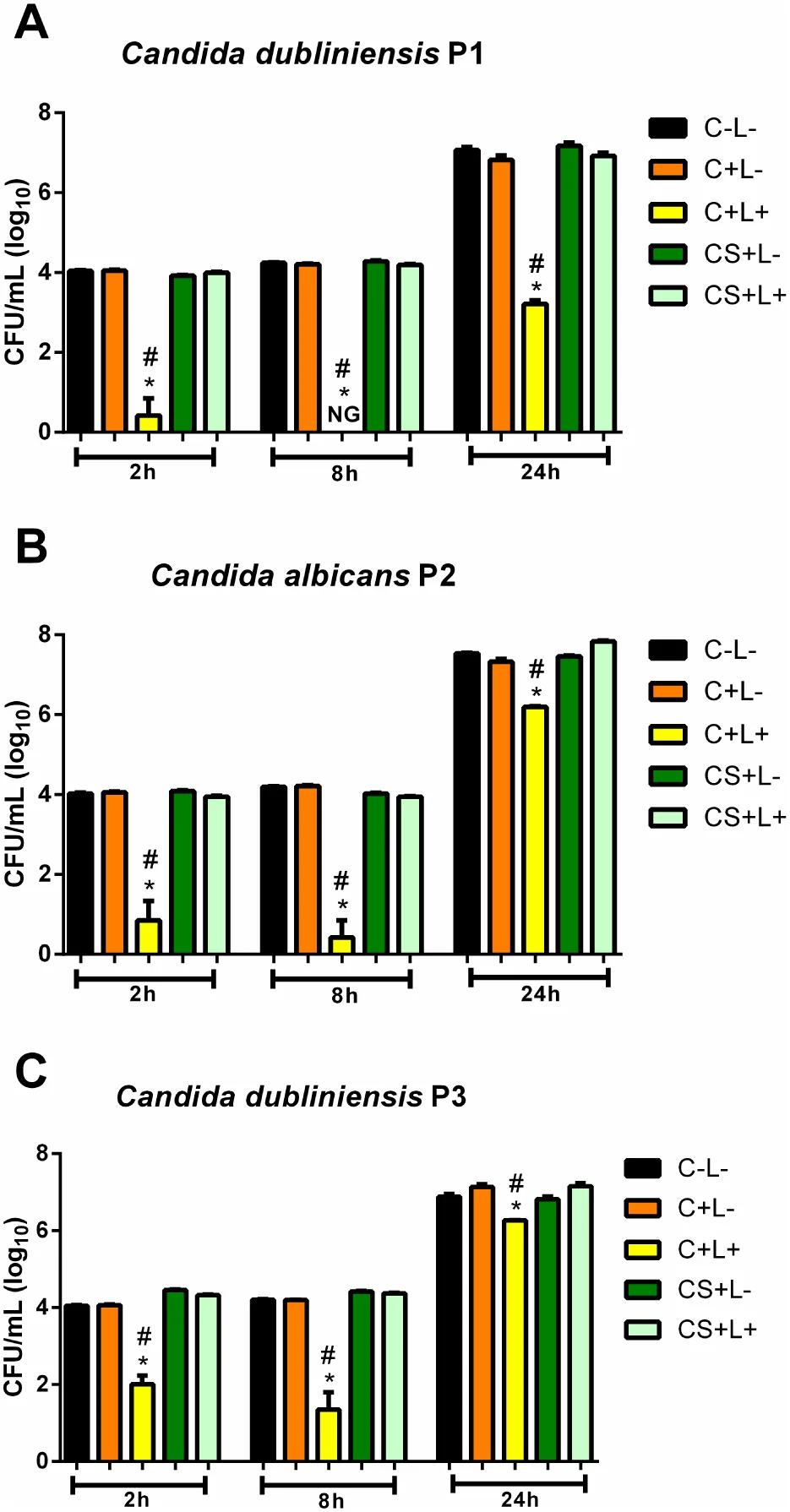
Viability (log_10_ CFU/mL) of A)*Candida dubliniensis*P1, B)*Candida albicans*P2, and C)*Candida dubliniensis*P3 not treated (C-L-) and treated with curcumin (CUR) without light (C+L-), curcumin-associated superparamagnetic iron oxide nanoparticles (CUR-SPION) without light (CS+L-), CUR with light (C+L+), and CUR-SPION with light (CS+L+) at 2, 8, and 24 h. NG: no growth. *: significant difference (*p* < 0.05) compared to the C-L- group. #: significant difference (*p* < 0.05) compared to the C+L- group.

### CUR-aPDT Reduced the Viability of Monotypic*Candida*Biofilms

The study of the impact of aPDT on monotypic biofilms of*Candida*spp. revealed a reduction in the viability of*C. dubliniensis*P1 in both the C+L- and C+L+ groups across all tested CUR concentrations. However, neither CUR-SPION without light nor CUR-SPION-mediated aPDT affected the biofilm viability at any of the CUR-SPION concentrations evaluated. In contrast, a significant reduction in the viability was observed in the C+L+ group, with decreases of 0.8 log_10_CFU/mL, 0.7 log_10_CFU/mL, and 1,2 log_10_CFU/mL at 1×, 5×, and 10× MIC, respectively. Treatment with light alone (C-L+) did not affect the viability of*C. dubliniensis*P1 (Figure 5A).

**Figure 5:**
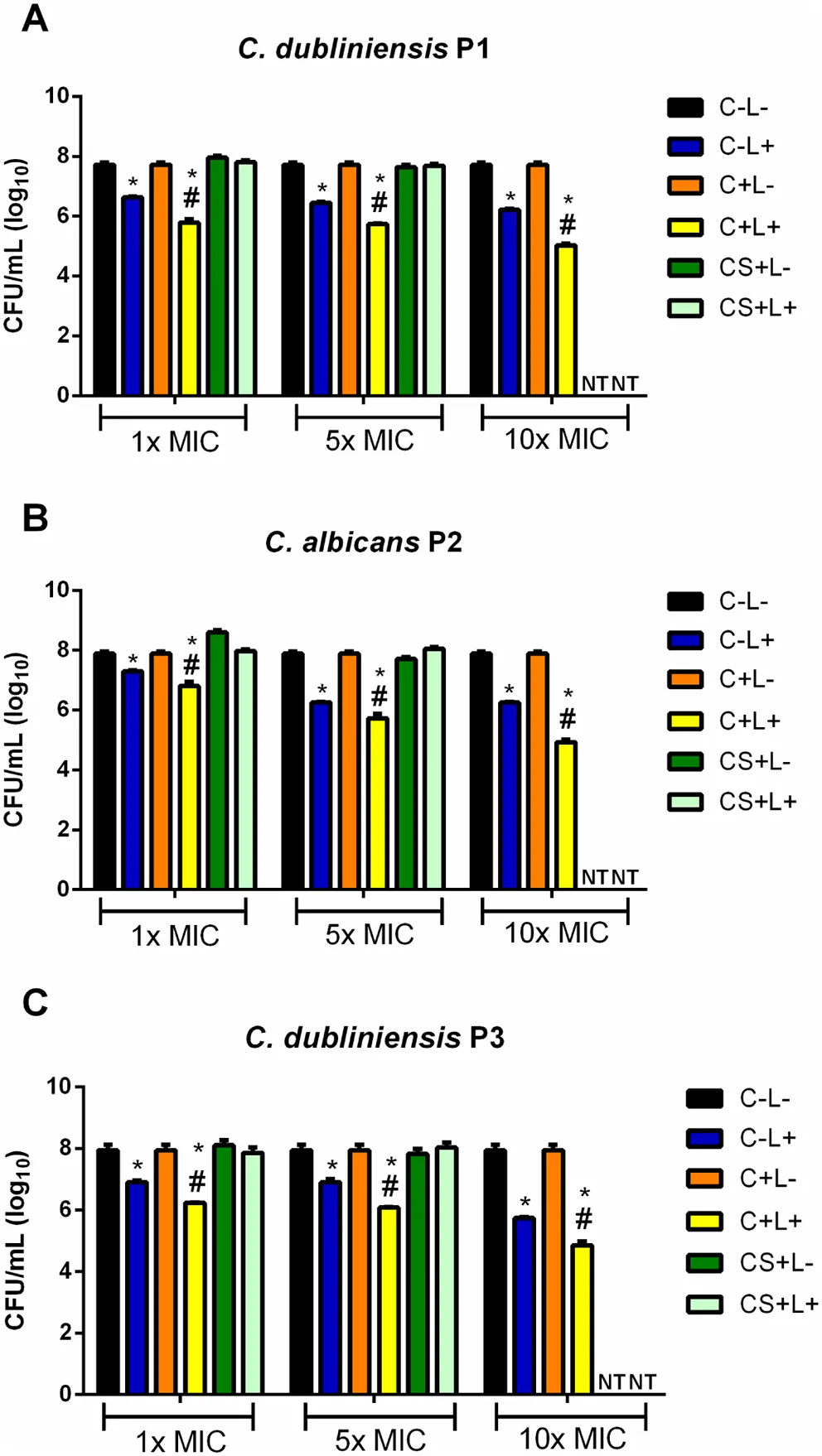
Viability (log_10_ CFU/mL) of monotypic biofilms of A)*C. dubliniensis*P1, B)*C. albicans*P2, and C)*C. dubliniensis*P3 not treated (C-L-) and treated with curcumin (CUR) without light (C+L-), curcumin-associated superparamagnetic iron oxide nanoparticles (CUR-SPION) without light (CS+L-), CUR with light (C+L+), and CUR-SPION with light (CS+L+). Concentrations of 1×, 5×, and 10× MIC of CUR, and 1× and 5× MIC of CUR-SPION were tested. *: significant difference (*p* < 0.05) compared to C-L- at the same MIC. #: significant difference (*p* < 0.05) compared to C+L- at the same MIC. MIC: minimum inhibitory concentration. NT: not tested.

Similarly, the viability of*C. albicans*P2 biofilms decreased at all concentrations in both the C+L- and C+L+ groups. However, irradiation (C+L+) markedly enhanced the antifungal activity of CUR, with reductions in the C+L+ group of 0.5 log_10_ CFU/mL at 1× MIC, 0.5 log_10_ CFU/mL at 5× MIC, and 1.3 log_10_ CFU/mL at 10×MIC. Exposure of*C. albicans*P2 biofilms to light (C-L+) did not impact fungal viability (Figure 5B).

Treatment with light (C-L+) did not affect the viability of the*C. dubliniensis*P3 biofilms. In contrast,*C. dubliniensis*P3 biofilm viability was significantly decreased following CUR-aPDT (C+L+) at 1× (0.7 log_10_ CFU/mL), 5× (0.9 log_10_ CFU/mL), and 10× MIC (0.9 log_10_ CFU/mL). Treatment with CUR without light (C+L-) caused a slight reduction in biofilm viability at all concentrations tested (Figure 5C).

### CUR-aPDT Decreased Microbial Viability in Microcosm Biofilms

Then, we investigated the effects of CUR-aPDT on microcosm biofilms of denture stomatitis. Given that CUR-SPIONs did not exhibit antifungal activity against*Candida*cells in prior assays, their effects on microcosm biofilms were not further evaluated.

In sample P1, CUR (C+L-) decreased microbial growth on BHI, MS, MSBS, MAN, and SDA. However, this antimicrobial effect was enhanced with CUR-aPDT, leading to a significant reduction in microbial counts in BHI (2.2 log_10_CFU/mL), MAN (1.5 log_10_ CFU/mL), MS (1.3 log_10_ CFU/mL), and MSBS (1.5 log_10_ CFU/mL) media compared to the C-L- group. However, light exposure (C+L+) did not potentiate the effects of CUR against yeasts (Figure 6).

**Figure 6:**
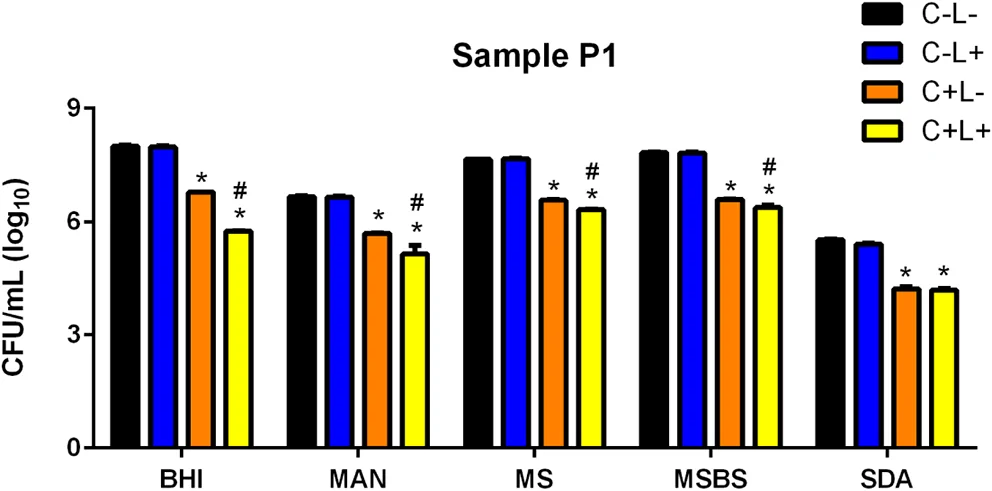
Microbial viability (log_10_CFU/mL) of microcosm biofilm from sample P1 not treated (C-L-), treated with light without curcumin (CUR; C-L+), CUR without light (C+L-), and CUR and light (C+L+), and plated on brain heart infusion (BHI) agar, mitis salivarius (MS) agar, mitis salivarius bacitracin sucrose (MSBS) agar, mannitol (MAN) agar and Sabouraud dextrose agar (SDA). *: significant difference (*p* < 0.05) compared to the C-L- group in the same culture medium. #: significant difference (*p* < 0.05) compared to the C+L- group in the same culture medium.

Microbial counts of the microcosm biofilm from sample P2 were also reduced after treatment with CUR (C+L-) on BHI, MS, MSBS, MAN, and SDA media. In the C+L+ group, the counts of streptococci (1.5 log_10_ CFU/mL), *mutans* streptococci (3.1 log_10_ CFU/mL), yeasts (3.1 log_10_ CFU/mL), and the nonselective medium (1.3 log_10_CFU/mL) were significantly reduced compared to the group treated with CUR without light (C+L-) (Figure 7).

**Figure 7:**
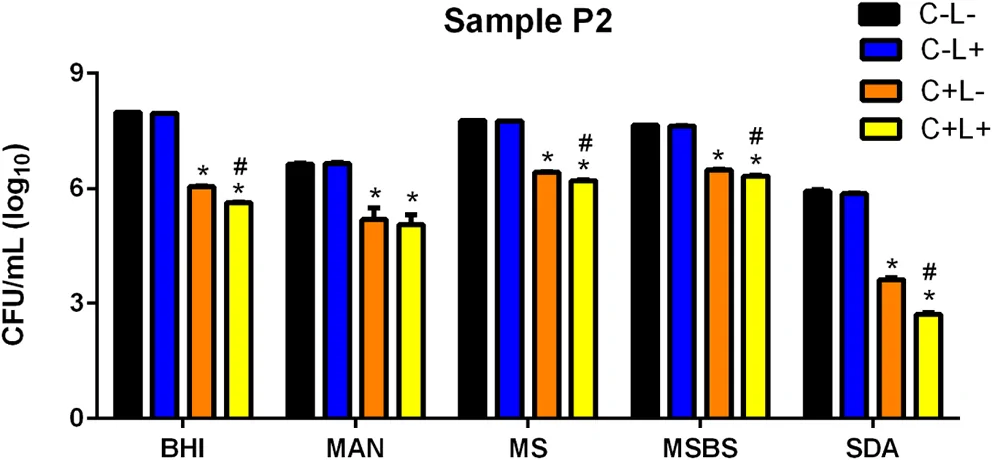
Microbial viability (log_10_CFU/mL) of microcosm biofilm from sample P2 not treated (C-L-), treated with light without curcumin (CUR; C-L+), CUR without light (C+L-), and CUR and light (C+L+), and plated brain heart infusion (BHI) agar, mitis salivarius (MS) agar, mitis salivarius bacitracin sucrose (MSBS) agar, mannitol (MAN) agar, and Sabouraud dextrose agar (SDA). *: significant difference (*p* < 0.05) compared to the C-L- group in the same culture medium. #: significant difference (*p* < 0.05) compared to the C+L- group in the same culture medium.

In sample 3, treatment with CUR without irradiation (C+L-) decreased microbial counts on the nonselective medium and reduced counts of streptococci, *mutans* streptococci, staphylococci, and yeasts. However, light potentiated the antimicrobial effects of CUR, resulting in reductions of 2.4 log_10_ CFU/mL in BHI, 2.2 log_10_ CFU/mL in MAN, 2.3 log_10_ CFU/mL in MS, 1.4 log_10_ CFU/mL in MSBS, and 1.2 log_10_ CFU/mL in SDA (Figure 8). Light alone (C-L+) did not affect microbial viability in any of the samples tested (Figures 6, 7, and 8).

**Figure 8:**
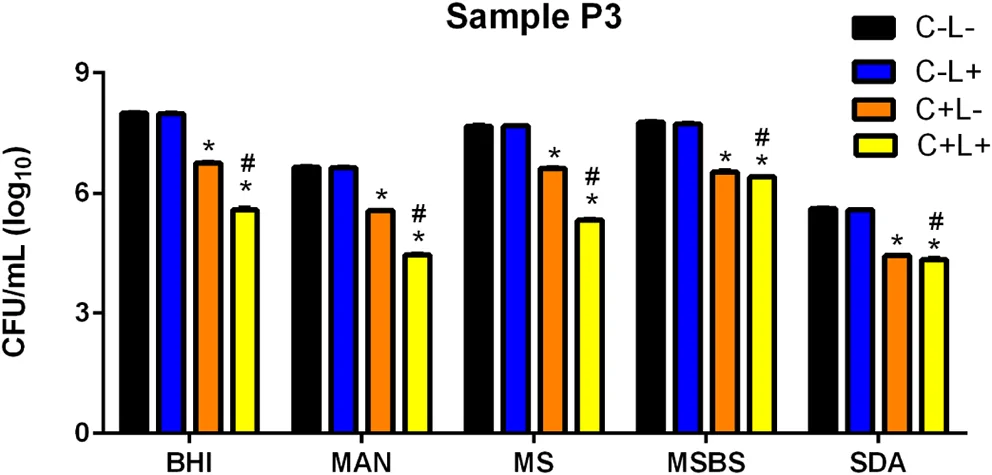
Microbial viability (log_10_CFU/mL) of microcosm biofilm from sample P3 not treated (C-L-), treated with light without curcumin (CUR; C-L+), CUR without light (C+L-), and CUR and light (C+L+), and plated brain heart infusion (BHI) agar, mitis salivarius (MS) agar, mitis salivarius bacitracin sucrose (MSBS) agar, mannitol (MAN) agar. and Sabouraud dextrose agar (SDA). *: significant difference (*p* < 0.05) compared to the C-L- group in the same culture medium. #: significant difference (*p* < 0.05) compared to the C+L- group in the same culture medium.

## Discussion

Denture stomatitis is a condition affecting mucosa supporting removable prostheses—either partial or total—and is clinically characterized by hyperemia, edema, congestion, and, in some cases, petechiae.^7^ This manifestation is commonly associated with*Candida*spp. infections.^5,6^ The recurrent nature of denture stomatitis and the limited therapeutic options available for managing*Candida*-associated cases have prompted the search for alternative treatment strategies. In this study, we investigated the antifungal effects of aPDT mediated by free CUR and CUR-SPION. To this end, microcosm biofilm samples associated with denture stomatitis were collected, followed by the isolation and identification of*Candida* species present in these samples.

First,*Candida*spp. was detected in samples obtained from patients clinically diagnosed with denture stomatitis, and species-level identification of these isolates was performed using MALDI-TOF MS. Two isolates were identified as*C. dubliniensis*and one as*C. albicans*. Then, we proceeded with stage 2 of the study. This phase focused on evaluating the effects of CUR and CUR-SPION as photosensitizers in aPDT on*Candida*planktonic cells and monotypic biofilms. CUR is a polyphenolic compound with broad antimicrobial activity, whose action can be enhanced through its use as a photosensitizer in aPDT.^28–30^ In addition to evaluating free CUR, we also synthesized CUR-SPIONs and assessed their antifungal effects as a photosensitizer in aPDT.

Characterization data confirmed the successful synthesis of CUR-SPION. In addition, FTIR analysis provided evidence of CUR incorporation into CUR-SPION; however, these results do not constitute unequivocal confirmation. The IR spectra of CUR show stretching vibrations at 1628 cm^–1^ attributed predominantly to the overlapping stretching vibrations of alkenes (C=C) and carbonyl (C=O) character. In addition, a C=C aromatic stretching vibration at 1427 cm^–1^ and a high-intensity band at 1512 cm^–1^ were attributed to the mixed vibrations including stretching carbonyl bond vibrations (C=O). Stretching vibration at 3200–3500 cm^–1^ is due to O–H groups. Furthermore, significant intense band at 1277 cm^–1^ is attributed to the bending vibration of the (C–O) phenolic band.^50,65^ Moreover, the characteristic peak of the Fe–O bond between the region of 440 and 600 cm^–1^ was associated with the intrinsic vibration of the tetrahedral and octahedral sites of the iron oxide crystal.^66^ In addition, zeta potential analysis confirmed good colloidal stability of CUR-SPION. The combination of characteristic CUR vibrational bands, modifications in the SPION vibrational patterns between 440–600 cm^–1^, alterations in the zeta potential following synthesis with CUR, and the morphological features observed in TEM images collectively support the incorporation of CUR into CUR-SPION. Nevertheless, additional quantitative analyses, such as high-performance liquid chromatography and thermogravimetry, are required to quantify CUR in CUR-SPION.

Despite successful synthesis, CUR-SPION did not exhibit activity against*Candida*planktonic cells or monotypic biofilms. Interestingly, this lack of effect was observed under both nonirradiated and irradiated conditions under the parameters applied in this study. Previous studies have reported promising results with iron-based nanoparticles against*Candida*spp. For instance, Seddighi et al.^38^ demonstrated the inhibitory effects of synthesized iron-oxide nanoparticles (IONPs) on *Candida*planktonic cells. Similarly, Khodavandi et al.^67^ reported that magnetic iron oxide nanoparticles (MIONs) inhibited*C. albicans*at 500 μg/mL, probably through increased ROS production and downregulation of biofilm-related genes *HWP1* and *ALS1*. Notably, their study showed that the antifungal activity of MIONs was enhanced in the presence of white light. It is possible that CUR-SPION formulation could yield promising results at higher concentrations. However, the synthesis strategy employed in this study resulted in a final concentration of 350 μg/mL, which limited to perform assays at levels comparable to those reported in previous studies in which iron-based nanoparticles demonstrated antifungal effects.^38,67^

In another study, de Santana et al.^37^ successfully synthesized CUR-SPIONs that, when applied in aPDT, exhibited inhibitory effects against*S. aureus*. A comparative analysis between their synthesis method and that employed in the present study suggests that the efficacy of CUR-SPIONs may be influenced by multiple factors, including the microbial target, the synthesis approach, and the specific aPDT parameters. Notably, ζ-potential analysis revealed that our synthesized CUR-SPION possesses a negative surface charge.*Candida*cells also exhibit a negatively charged surface because of structural polysaccharides in the cell wall. This similarity in charge leads to electrostatic repulsion, thereby hindering the interaction between CUR-SPION and*Candida*cells.^68^ However, further studies are necessary to confirm this hypothesis. In addition, we believe that modifications to the synthesis methodology or the implementation of surface engineering strategies could potentially alter the physicochemical properties of CUR-SPION and increase the concentration of the colloidal suspension, thereby enhancing their antifungal efficacy.

It is important to note that different synthesis methods can directly impact the antifungal efficacy of the nanoformulations. For example, the CUR-SPION successfully synthesized by de Santana et al.,^37^ which demonstrated antibacterial effects, was produced using a conjugation method in which covalent bonds were formed between CUR and the surface of SPION. Moreover, studies have shown that the conjugation of photosensitizers, such as porphyrins, phthalocyanines, and chlorins, with SPIONs decreases nanoparticle aggregation, increases their stability, and subsequently enhances their therapeutic potential.^41–43^ Coating strategies, characterized by the deposition of a layer of CUR or CUR-containing material onto previously formed nanoparticles through noncovalent interactions, have also been explored, primarily for bioimaging and cancer therapy.^69–71^ The use of SPIONs in these therapies has been associated with their superparamagnetic properties, which provide excellent contrast for magnetic resonance imaging, and with their ability to induce magnetic hyperthermia when exposed to a magnetic field of specific frequency. This process leads to heating and death of cancer cells.^69,70,72,73^ However, there are no data regarding the antimicrobial effects of the nanoformulations.

In our study, we employed an association approach during SPION synthesis, in which CUR was present during coprecipitation,^49,52^ allowing its incorporation and association with nanoparticles during their formation. This difference in the synthesis approach may influence the antimicrobial effects of CUR-SPIONs. Nevertheless, new synthesis strategies should be investigated.

Since CUR-SPION did not exhibit activity in the conditions studied, we investigated the effects of CUR-mediated aPDT on*Candida*planktonic cells,*Candida*biofilms, and microcosm biofilms. Irradiation significantly enhanced the antifungal effects of CUR, reducing MIC values by 16-fold and highlighting CUR’s use as a photosensitizer in aPDT. CUR is a natural, low-cost photosensitizer capable of absorbing light in the blue spectrum, which likely increases ROS production and contributes to its antimicrobial effects.^25,74,75^ Furthermore, CUR-aPDT decreased planktonic cell viability at 8 h; however, an intriguing increase in fungal growth was observed 24 h post-treatment. The fungistatic mechanism of CUR used in aPDT against*Candida*spp., as previously reported by Casu,^76^ may explain this regrowth. Despite this finding, fungal growth remained significantly lower than in cells treated with CUR without light. It is important to note that, within the host, the fungistatic activity of CUR-mediated aPDT may act synergistically with the immune system response, potentially contributing to the reduction or resolution of the infection.

Therefore, we investigated the effects of CUR-mediated aPDT on monotypic*Candida*biofilms. Biofilms are communities of microorganisms embedded in a matrix of extracellular polymers on biotic or abiotic surfaces, including oral mucosa and dentures.^77^ In this study, CUR-mediated aPDT at 4 μg/mL reduced the viability of monotypic biofilms of*C. dubliniensis*P1 and*C. dubliniensis*P3 by 25.04% and 21.53%, respectively. However, greater reductions were observed after aPDT with CUR at 40 μg/mL. Sanitá et al.^78^ demonstrated that CUR-mediated aPDT affected the viability of*C. dubliniensis*biofilms in a species-dependent manner, reducing biofilm viability at least 57.70% at 8 μg/mL. Another study reported that CUR-aPDT at 22 μg/mL decreased the viability of*C. albicans*biofilms by 66.44% to 90.87%.^32^ In this study, the reductions in*C. albicans*biofilms ranged from 13.64 to 37.56% after CUR-aPDT at 4 and 40 μg/mL, respectively. These differences in antifungal effects could be attributed to variations in the CUR source,*Candida*strains, biofilm formation strategies, and aPDT parameters used in different studies.

Although the effects of CUR-mediated aPDT against cells and monotypic biofilms of*Candida*are well established,^31,76,78,79^ there are limited studies evaluating its efficacy on complex and heterotypic biofilms. In addition, no studies to date have explored the use of CUR in aPDT specifically for treating microcosm biofilms of denture stomatitis. The microcosm biofilm model represents the complexity of *in vivo* biofilms, which are characterized by microorganisms from various species and, in some cases, different kingdoms, such as bacteria and fungi.^22,80^ In a previous study, Carmo et al.^64^ investigated the antimicrobial effects of the polyphenol pterostilbene against denture stomatitis microcosm biofilms. Although aPDT was not employed, treatment with pterostilbene reduced the viability of both bacteria, including*Streptococcus*and *Staphylococcus*, as well as*C. dubliniensis*at 160 μg/mL. Interestingly, concentrations of 640 μg/mL were required to reduce the viability of*C. albicans*within these complex and heterotypic microcosm biofilms.

In this study, CUR-mediated aPDT at 40 μg/mL significantly reduced microbial viability (nonselective counting) of microcosm biofilms associated with denture stomatitis. Notably, the counts of streptococci and *mutans* streptococci were reduced in samples P1, P2, and P3.*Streptococcus*spp. are the primary colonizers of oral surfaces, constituting over 70% of the cultivable bacteria in dental biofilms. Streptococci from *mutans* group, a group of Gram-positive, fermentative, and acidogenic bacteria, can produce extracellular polysaccharides and thriving in acidic environments. Due to their strong adhesion to dental structures, *mutans* streptococci are recognized as a leading cause of dental caries, particularly in fissures and smooth surfaces.^81–83^

The viability of*Staphylococcus*was reduced in samples P1 and P3. Staphylococci are part of the oral microbiota, with *S. aureus*being the primary pathogenic species of the genus, commonly found in the oral cavity of healthy adults and dental prosthesis users.^29,84^ Additionally, staphylococci are associated with oral infections such as angular cheilitis and staphylococcal mucositis.^21,85,86^ CUR-mediated aPDT demonstrated antibacterial effects against planktonic *S. aureus*, with significant reductions observed at 50 μg/mL. However, higher concentrations were required to decrease the viability of monotypic*S. aureus*biofilms.^87^ In this study, reductions in staphylococci within microcosm biofilms were achieved following CUR-mediated aPDT at 40 μg/mL. These findings demonstrate that, under the tested parameters, CUR-aPDT effectively reduced the viability of pathogenic bacteria in microcosm samples, including streptococci and staphylococci.

Yeast counts from samples P2 and P3 were also reduced after the CUR-aPDT treatment.*Candida*spp. play a key role in the pathogenesis of denture stomatitis, primarily due to its ability to form biofilms on abiotic surfaces, including dual-species biofilms with*S. aureus*and*S. mutans*.^33,88^ Interestingly, CUR-aPDT effectively reduced the counts of two*Candida*species (*C. albicans* and*C. dubliniensis*), highlighting its broad-spectrum antimicrobial effects. Indeed, the treatment of dual-species biofilms formed by*S. mutans*and*C. albicans*with CUR resulted in decreased matrix extracellular polysaccharide production and downregulation of quorum sensing-related gene expression in*S. mutans*,^33^ reinforcing the multitarget mode of action of CUR and CUR-aPDT against interkingdom biofilms.

In summary, this study demonstrated that CUR-mediated aPDT effectively targeted fungal cells and monotypic biofilms of *C. albicans*and*C. dubliniensis*isolated from denture stomatitis samples. More importantly, CUR-mediated aPDT significantly reduced the viability of pathogenic bacteria and yeasts within the samples. These findings have the potential to impact dentistry for several reasons. First, they demonstrate the feasibility of using a photosensitizer activated by blue light, enabling the use of a photopolymerizer—a device commonly available in dental clinics. Second, the protocol involves a short preirradiation time (5 min), which enhances its practicality in clinical settings. Third, the study analyzes samples from patients with denture stomatitis associated with*Candida*spp., a condition that presents therapeutic challenges because of limited treatment options and a high recurrence rate. However, the effects of this strategy need to be investigated using a larger number of samples from denture stomatitis and various*Candida*species, including non-*albicans* strains.

Regarding the absence of antifungal effects of CUR-SPION both under nonirradiated and irritated conditions, we hypothesize that modifications to the synthesis strategy could produce nanoparticles with higher concentrations and a positive surface charge, potentially enhancing their antifungal efficacy.
